# Nitrous oxide occupational exposure in conscious sedation procedures in dental ambulatories: a pilot retrospective observational study in an Italian pediatric hospital

**DOI:** 10.1186/s12871-019-0714-x

**Published:** 2019-03-27

**Authors:** S. Zaffina, M. Lembo, F. Gilardi, A. Bussu, F. Pattavina, M. G. Tucci, U. Moscato, M. Raponi, P. Derrico, A. Galeotti, V. Camisa

**Affiliations:** 1 Occupational Medicine, Health Directorate, Bambino Gesù Children’s Hospital - IRCCS, Piazza di Sant’Onofrio 4, 00165 Rome, Italy; 2 Risk Management and Technology Assessment Department, Bambino Gesù Children’s Hospital, IRCCS, Rome, Italy; 3 Section of Hygiene, Institute of Public Health, Fondazione Policlinico Universitario A. Gemelli IRCCS, Roma - Università Cattolica del Sacro Cuore, Rome, Italy; 4 Health Directorate, Bambino Gesù Children’s Hospital, IRCCS, Rome, Italy; 5 Dentistry Unit, Department of Pediatric Surgery, Bambino Gesù Children’s Hospital, IRCCS, Rome, Italy

**Keywords:** Nitrous oxide, Occupational exposure, Conscious sedation, Risk assessment, Dentistry

## Abstract

**Background:**

Nitrous oxide has a proven clinical efficacy in conscious sedation. At certain environmental concentrations it may pose a health risk to chronically exposed healthcare workers. The present pilot study aims at evaluating the exposure to nitrous oxide of dental ambulatory personnel of a pediatric hospital.

**Methods:**

A descriptive study design was conducted in two phases: a bibliographic analysis on the environmental safety policies and a gas concentration analysis in the dental ambulatories of a pediatric hospital, detected every 6 months from December 2013 to February 2017 according to law provisions.

The surveys were carried out using for gas analysis a photoacoustic spectrometer Innova-B&K “Multi-gas monitor model 1312” and Innova-B&K “Multi-sampler model 1309”. The biological analysis and monitoring have been carried out on staff urine.

**Results:**

The analyses were performed during 11 dental outpatient sessions on pediatric patients. All the patients were submitted to the same dental procedures, conservative care and dental extractions. The pediatric patients were 47 (23 males, 24 females; age range 3–17 years; mean age 6,63, SD ± 2,69) for a mean of 4,27 (SD ± 1,49) per session., The mean environmental concentration of nitrous oxide during the sessions was 24.7 ppm (SD ±16,16). A correlation was found between urinary nitrous oxide concentration of dentists (Pearson’s correlation 0.786; *p* = 0.007) and dental assistants urines (Pearson’s correlation 0.918; *p* < 0.001) and environmental concentrations of nitrous oxide. Weak negative correlations were found between age and sex of patients and environmental concentrations of nitrous oxide. The mean values of the biological monitoring data referring to all the outpatient sessions are lower than the reference values foreseen in accordance to the regulations in force on nitrous oxide concentration.

**Conclusions:**

The mean environmental concentration values recorded in our study are below the limit of 50 ppm considered as a reference point, a value lower than those reported in other similar surveys. The results of the present study provide a contribution to the need to implement technical standards, criteria and system requirements for the dental ambulatories, to date not yet completely defined, and cannot be assimilated to the ones established for the surgical rooms.

## Background

The term *conscious sedation* refers to that “*pharmacological technique that induces patients in a state of depression of the central nervous system in which they are able to respond appropriately and rationally to physical stimulation and verbal command for the entire period of treatment”* [1].

As demonstrated in several scientific studies, this technique is widely used also in the dental field, facilitating pain tolerance and anxiety control, allowing maintenance of the state of consciousness and reduction of the perioperative adverse events in patients suffering from chronic diseases [2–11]. In this context the use of nitrous oxide [12] is particularly suitable to pediatric patients [11–13]. Conscious inhalation sedation with nitrous oxide and oxygen is the gold standard in outpatient dentistry practice, for the rapid establishment and the equally rapid exhaustion of the sedative effect [11].

Nitrous oxide has demonstrated a proven clinical efficacy but, at certain environmental concentrations, may pose a health risk to chronically exposed medical personnel. Literature reports several studies that associate chronic exposure to nitrous oxide with the onset of adverse effects also on personnel exposed to dental practice. The most common reported side effects are: increase in the rate of spontaneous abortion, infertility and reproductive difficulties, congenital anomalies and delay of fetal growth; increase in the incidence of cancer in the uterine cervix and kidney, liver diseases; adverse effects on bone marrow function and immune system, generalized neurological disorders and psychomotor impairment [14–18].

One of the main questions in dental care about the conscious sedation with nitrous oxide is connected with the definition of the reference values in the dental ambulatory practice, not comparable with the ones established for the surgical rooms.

The current and applicable reference values of concentration of nitrous oxide and the calculation of efficacious air changes applicable to dental ambulatories in which the gas under study is being used, are in fact a specific issue to be defined on the basis of national and international literature and policies.

The objective of the study, besides assessing the personnel occupational exposure, aims at supporting the scientific community in identifying the reference environmental values specific for dental ambulatories that make use of nitrous oxide, with a specific regard to the pediatric context.

## Methods

### Study design

A descriptive study design was conducted in two phases: a bibliographic analysis on the environmental safety policies and a gas concentration analysis in the dental ambulatories of a pediatric hospital, performed every 6 months from December 2013 to February 2017, according to law provisions.

STROBE Statement—checklist of items of observational studies has been included in drafting the present article.

### Setting

The environmental and biological analyses were carried out in two dental ambulatories located in two different sites of the Dentistry Unit, Department of Pediatric Surgery, Bambino Gesù Children’s Hospital, IRCCS, Rome Italy. The two dental ambulatories consist of one room and one dental unit each one.

Over the course of the last 3 years the following are the numbers of cases treated by the Dentistry Unit of the hospital (Table 1).

**Table 1:** Cases treated by the Dentistry Unit in the last 3 years

Site	2015	2016	2017
1	476	386	466
2	405	502	372

### Bibliographic analysis of national and international literature and policies

In absence of specific provisions, a bibliographic research was carried out in order to identify and compare the current and applicable reference values of concentration of nitrous oxide in dental ambulatories.

Moreover, through the analysis of the national and international legislation and the calculation of efficacious air changes, with this study the authors tried to identify and propose a unique reference value for the minimum number of air changes/hour, applicable to dental ambulatories in which the gas under study is being used.

### Participants

#### Dental ambulatory team for procedures

The team that works in the dental ambulatory during each surgical session is composed by: one dentist and one dental assistant.

#### Personnel involved in environmental measuring

The personnel required for the environmental analysis and the collection of urinary samples consists of an operator with the qualification of prevention technician. The analysis of the environmental data and the urinary samples was performed at accredited laboratories.

#### Selected personnel for the sample

Personnel involved in urinary nitrous oxide sampling is the staff on duty during the dental session selected for monitoring. The staff is invited to participate in the collection of the biological samples within the occupational health prevention program. The results of the biological monitoring on the single operator are stored in the personal health record of the Occupational Medicine Unit.

#### Pediatric patients

The pediatric patients subjected to dental procedures in the 11 sessions were 47 (23 males, 24 females) with an age range from 3 to 17 years.

### Variables

The main variables of the study are:

- as for the HCWs involved: job category, environmental and urinary nitrous oxide concentration
- as for the patients involved: gender and age

All the variables were treated as quantitative variables. Gender was considered as a percentage of males for each session.

### Data sources and measurement

#### Methods and administration systems

Inhalation sedation was administered with the Master Flux Plus (Tecno-gaz, Parma, Italy) apparatus which allows the administration of a mixture of oxygen and nitrous oxide, with a ratio ranging from 0:100 to 70:30 in favor of nitrous oxide, with the aim of obtaining an analgesic and slightly sedative effect on pediatric patients. The health operator can choose the treatment of the gases exhaled by the patient in two ways: a discharge through a surgical suction device of the gases directly from the mask or a gas discharge on the outside.

In the initial phases of the intervention sessions, a percentage of 20% nitrous oxide was administered, which was then increased according to the reaction observed in the patient. The flow of nitrous oxide mixture was around 4–5 l/min. At the end of the dental session the patient was given only oxygen, at a flow of 2–3 l/min for 2 min, then the patient was required to stay in the waiting room for about 5–10 min and, finally, discharged.

The device consists of: a 2.7-l rubber flask attached to a flow-metric box on which there is a button that allows the filling of the rubber ball with oxygen, to minimize possible toxic effects due to nitrous oxide; an exhausted gas evacuation filter; a tube for the evacuation of gas; a nasal mask. The mixture of nitrous oxide with oxygen can be administered through the use of a mask connected with a mobile evacuation system of the gases expired by the patient that allows a rapid and effective action, thus reducing the environmental dispersion of nitrous oxide and, therefore, HCWs exposure.

#### Instrumental measures

The present study was carried out at the Dentistry outpatient ambulatories of a large national pediatric hospital.

The primary outcome measurements of the study are related to the assessment of nitrous oxide environmental and biological concentrations during the dental pediatric session. These measurements are performed in order to control the level of HCWs exposure to the anesthetic gas and test the efficacy of the prevention system (structure/implant characteristics, anesthesia equipment, work procedures and human factors).

The environmental concentrations of nitrous oxide were detected using an instrument during the half-yearly surveys in the period December 2013–February 2017 for a total of 11 surveys. The instrument used for gas analysis is the photoacoustic spectrometer Innova-B&K (Brüel & Kjær, Nӕrum, Denmark) **“**Multi-gas monitor model 1312” and Innova-B&K (Brüel & Kjær, Nӕrum, Denmark) “Multi-sampler model 1309”.

Measurements were performed using the instrument for the whole outpatient session, for a minimum of 4 h. The measuring probes were placed in the dentist’s operating area, at a height between 120 and 150 cm from the ground (at the level of the breathing area zone), in order to detect the exposure conditions and not be an obstacle to the health operator sitting next to the patient.

The biological samples were taken at the beginning and at the end of the working shifts of the personnel involved; biological monitoring was carried out on their urine for the biological analysis. Biological monitoring was performed by taking urine samples from the dentist and dental assistants at the beginning and at the end of the working activity. The samples were then analyzed in laboratory, by means of a Thermo Trace GC Ultra with Polaris Q Mass Spectrometer GC/MS System (IET-International Equipment Trading Ltd., Mundelein, Illinois, USA), and Thermo Direct Probe Controller (IET-International Equipment Trading Ltd., Mundelein, Illinois, USA).

The mean values of the biological monitoring data referring to all the outpatient sessions were controlled with the reference values foreseen in relation to the regulations in force on nitrous oxide. The air changes/hour were calculated by measuring the air flow rate in m^3/^h of the diffusers present in each room with an TSI 8375 Accubalance Capture Hood (TSI, Shoreview, Minnesota, USA) flow conveyor. The Ambient Theoretical Air changes were calculated, by using a propeller anemometer Testovent 417 (Testo Spa, Settimio Milanese, Milano, Italy) measuring the flow rate of the anemostats, both for retake and intake, present in the dental ambulatory under study. The air flows were measured with the anemometer placed in contact with each retake and intake grid, placed on the direct air flow. The parameters were calculated using the formulas indicated by the international standards [19] and the “Istituto Superiore per la Prevenzione e la Sicurezza del Lavoro - ISPESL (High Institute for the Prevention and Safety of Work) 2009” Guidelines [20]. In addition, the number of effective air changes was calculated by constructing the tracer decay curve (nitrous oxide), plotting the natural logarithm (ln) of the tracer concentration in accordance to time.

The secondary outcome measurements of the study regard the evaluation of air changes in order to propose a specific reference value for conscious sedation in dental ambulatory settings.

#### Handling protocol of urine samples

Sampling of nitrous oxide in biological fluids (urine) was carried out exclusively on the HCWs present in the Dentistry Unit, appointed by the Health Directorate and the Surveillance Service.

Sample collection was performed in the following ways:

1. Time T_0_ = The operator emptied the bladder before entering the ambulatory at the beginning of his/her work shift filling the urine collection container provided by the hospital’s referent and handed over the sample leaving own personal data (name, surname and work assignment).
2. Time T_1_ = After a minimum of 4 h of exposure the operator urinated by filling up the urine collection container provided by the referent of the hospital and handed the sample to the operator in charge.

The operator who had finished his/her shift and/or had to move away from the dental ambulatory before the end of the 4-h observation period, is however requested to urinate as indicated in point 2 before leaving the dental ambulatory.

An immediate transfer of a urine aliquot to a hermetically sealed tube was therefore performed very quickly (t < l minute) so that the vapors loss was negligible (< 5%). The urines were then acidified with sulfuric acid (200 μl of H2S04 9 N as antimicrobial agent) and stored for more than 24 h. Collection was performed in environments free of pollution due to anesthetic gases.

The vials, appropriately labeled, were transported to the laboratories, through a thermostatic thermal bag and stored at 4 °C up to the analysis. The analysis was carried out in the laboratory using a gas chromatograph mass-type mass spectrometer Thermo Trace GC Ultra with Polaris Q Mass Spectrometer GC/MS System, and Thermo Direct Probe Controller.

### Bias

No specific bias of selection could be recognized. The choice of each ambulatory dental session is random and the personnel involved is individually controlled by the Health Directorate and Surveillance Service of the hospital with no predetermined reasons or conditions except for the necessities determined by the established criteria connected with the HCWs turnover workshifts.

All the procedures established for the management of nitrous oxide measurements, both environmental and biological, are strictly controlled by the competent personnel.

### Study and sample size

The study size comprehends the environmental and biological measurements of nitrous oxide of 11 pediatric dental ambulatory sessions. Eleven environmental measurements of nitrous oxide concentrations were performed, as previously described, and 21 HCWs were involved in the study divided into 10 dentists (the urinary sample of the dentist of one session is missed) and 11 dental assistants for the biological measurements of nitrous oxide concentrations. The pediatric patients subjected to dental procedures performed in the 11 sessions were 47 (females 51%). The sample size was determined by the number of HCWs and patients involved in the 11 dental ambulatory sessions that were examined twice a year from December 2013 to February 2017 for environmental and biological monitoring, as required by the Italian laws currently in force**.**

### Statistical methods

The collected data were entered into a database of the Excel program and analyzed using the Statistical SPSS Statistics software (IBM SPSS Statistics V20, Chicago, IL, USA). A descriptive analysis of the values of environmental and urine concentration of nitrous oxide is reported as Mean ± Standard Deviation. A correlation analysis between patients’ characteristics (age and gender) and environmental gas concentrations, dentist and dental assistant urine concentrations and environmental gas concentrations were performed. Missing data were excluded from the analysis.

## Results

### Environmental and biological monitoring

Eleven dental outpatient sessions, carried out on pediatric patients, have been monitored from December 2013 to January 2017.

### Descriptive data. Patients and dental procedures

The pediatric patients were 47 (23 males, 24 females; age range 3–17 years mean age 6.62, SD ± 2.69) for a mean of 4,27 (SD ± 1.49) per session. Non significant weak negative correlations were found between patients characteristics (age and gender) and environmental concentrations of nitrous oxide (Age: Pearson’s correlation = − 0.107; *p* = 0.51. Gender, Percentage of males per session: Pearson’s correlation = − 0.221; *p* = 0.75) All the patients were submitted to the same dental procedures, conservative care and dental extractions.

The percentage of males per session ranges from 0 to 66.7 (Table 2).

**Table 2:** Patients characteristics

Site	Date	Number of Patients	Gender	Age
1	05.12.13	4	2 M^−2^ F	5–12
1	11.06.14	2	2 F	8–17
1	17.11.14	3	2 M^−1^ F	5–11
1	12.06.15	3	2 M^−1^ F	5–8
2	02.07.15	3	2 M-1 F	5–8
1	26.11.15	4	2 M-2 F	4–7
2	18.01.16	7	3 M-4 F	3–6
2	28.07.16	5	1 M-4 F	4–12
1	29.07.16	6	4 M-2 F	3–10
2	16.01.17	5	3 M-2 F	5–8
1	17.02.17	5	2 M-3 F	5–7

### Outcome results

#### Environmental measures

The mean environmental concentration of nitrous oxide registered during the various outpatient sessions is shown in Table 3.

**Table 3:** Nitrous oxide environmental measurements in ppm

Site	Date	Min	Max	Mean	SD
1	05.12.13	0.65	127.92	17.06	32.22
1	11.06.14	0.94	288.00	15.74	38.66
1	17.11.14	1.08	147.00	25.75	35.65
1	12.06.15	0.94	288.00	15.74	38.66
2	02.07.15	1.05	83.10	5.51	10.37
1	26.11.15	1.17	274.00	30.1	57.50
2	18.01.16	1.14	194.00	13.9	21.00
2	28.07.16	1.18	40.30	8.19	8.68
1	29.07.16	1.10	99.90	33.3	31.70
2	16.01.17	1.17	369.00	39.7	63.00
1	17.02.17	1.11	215.0	60.9	69.00
Mean Values		1.05	193.29	24.17	

The mean environmental concentration of nitrous oxide reported in the 11 outpatient sessions investigated was 24.17 ppm (SD ±16.16). The mean minimum value recorded in the sessions was 1.05 ppm (SD ± 0..3); the mean maximum value was 193.29 ppm (SD ± 103.19) and the range was 192.24. Differences were recorded in the comparison between the two sites where the analyses were performed. In site 1 the mean environmental concentration recorded in seven out of eleven total sessions was 28.35 ppm while in site 2, in four out of eleven sessions, it was 16.83 ppm.

#### Biological monitoring

The biological monitoring data refer to the 11 outpatient sessions and are reported in Table 4.

**Table 4:** Urinary nitrous oxide concentration in μg/l

Site	Date	Dentist	Assistant
1	05.12.13	8.26	6.89
1	11.06.14	5.35	4.31
1	17.11.14	12.6	9.43
1	12.06.15	*	7.89
2	02.07.15	2.87	2.54
1	26.11.15	8.47	6.23
2	18.01.16	4.35	6.56
2	28.07.16	4.26	4
1	29.07.16	11.21	8.34
2	16.01.17	16.6	18.7
1	17.02.17	25.4	15.6
Mean Values		9.94	8.23

*missing

The mean urinary dentist concentration was 9.94 μg/l (SD ± 6.92). The mean urinary dental assistant concentration was 8.23 μg/l (SD ± 4.90). One missing data about the dental assistant’s urinary concentration was relieved and excluded from the analysis. The minimum value recorded in dentists was 4.26 μg/l and the maximum 25.4 μg/l. The minimum value recorded in dental assistants was 2.54 μg/l and the maximum 18.7 μg/l. A correlation was found between urinary nitrous oxide concentration of dentist (Pearson’s correlation = 0.786; *p* = 0.007) and dental assistant (Pearson’s correlation = 0.918; *p* < 0.001) and environmental concentrations of nitrous oxide. Differences were recorded between the two sites in the comparison of urinary concentration of dentists and dental assistants. The mean value in site 1 (measured on six out of eleven sessions) in dentists was 11.88 μg/l and 7.02 μg/l in site 2 (measured on four out of eleven sessions). A minor difference was recorded in dental assistants where the mean value in site 1 (measured on seven out of eleven sessions) was 8.38 μg/l and 7.95 μg/l in site 2 (measured on four out of eleven sessions).

#### Air changes

The number of air changes/hour of the dental ambulatories subject to measurements were:

- Site 1 (15.0 V/h)
- Site 2 (15.5 V/h)

### Main results

The mean values recorded during the study are lower than the reference ones foreseen in the regulations in force on environmental and urinary nitrous oxide concentration.

## Discussion

As for the results of the bibliographic analysis of national and international literature and policies, at a regulatory level, since there is no limit value of nitrous oxide specific for the outpatient settings, reference is made to the environmental value of 50 ppm foreseen by the Italian Ministerial Memorandum no. 5/1989 for operation theatres built after 1989. According to the current legislation, this value does not constitute a limit value, but only a reference index. Similarly, as for the nitrous oxide concentration in urine, the same Memorandum reports three different reference values: 13 μg/l, 27 μg/l and 55 μg/l of N2O in end-of-turn urine (equal, respectively, to an average exposure of 25 ppm = 45 μg/l, 50 ppm = 90 μg/l and 100 ppm = 180 μg/l) [21]. Moreover, the following are other international recommendations on gas concentrations:

- The American Conference of Governmental Industrial Hygienists (ACGIH) has assigned a Threshold Limit Value (TLV) of 50 ppm (90 mg/m3) as a Time-Weighted Average (TWA) for a normal 8-h workday and a 40-h workweek [22].
- The National Institute for Occupational Safety and Health (NIOSH) Recommended Exposure Limit (REL): 25 ppm, 46 mg/ m^3^ TWA over the time of exposure [23].
- The International Chemical Safety Cards (ICSC) International Standards specifically warn that exposure should be avoided by pregnant women [24].

At national level a normative reference is the Decree of the Executive Committee N. 52/R of 8/10/2008, Annex B of the Tuscany Region, Italy, on “Organizational, structural and technological, general and specific requirements and conditions for starting and operating dental ambulatories subject to authorization” [25]. It is a normative reference that specifically addresses the topic “conscious sedation with anesthetic gases”, with particular reference to the dental field and provides, for this type of activity, a minimum number of air changes equal to *N* ≥ 15 V/h. As reported in literature, this is considered the optimal value [26]. At national level, some regional authorities indicate a variable limit value depending on whether it refers to an operating room or outpatient activity. The Lazio Region, Italy, where the hospital is located, assimilates this latter activity to the outpatient surgery defining a minimum limit of air exchange equal to *N* ≥ 6 V/h [27].

At international level the regulatory framework and the guidelines are more precise. In fact, NIOSH, in the document “Assessment of Nitrous Oxide Exposure in a Pediatric Dentistry”, provides that in cases where anesthetic gases are used and where surgical procedures are carried out, the most suitable ventilation criteria are those required for the operating theatres [28]. Conversely, the Academy of Architecture for Health (AIA), in the document “Guidelines for Design and Construction of Hospitals and Health Care Facilities”, indicates for the operating theatres a minimum value of 15 V/h of total ventilation with a minimum of 3 V/h of external air [29]. Conversely, the American Society of Heating, Refrigerating and Air-Conditioning Engineers (ASHRAE) recommends for the operating theatres a minimum of 20 V/h of total ventilation with a minimum of 4 V/h of outdoor air [30]. Finally, both AIA and ASHRAE state that an evacuation system is required when anesthetic gases are used. Another reference is the Standard 62 of the American National Standards Institute (ANSI)/ASHRAE: *Ventilation for Acceptable Indoor Air Quality* [31], which provides the requirements for outdoor air suitable for ventilation in the health facilities and which fixes the requirements in both health facilities and operating theatres at 50.97 m^3^/h.

As for the proposed objective of the study, the key results of the present research show that, even if in some single dental procedures the exposure of personnel to nitrous oxide was higher than 50 ppm as the maximum detected value, during all the days in which monitoring was carried out, its mean concentration remained below the limit established by the Addendum No. 5 of the Italian Ministry of Health of 1989. In this study, all dental treatments were successfully completed, thus also confirming the clinical efficacy of this type of sedation in the management of anxiety in children, as already highlighted by previous surveys [13]. Furthermore, previous studies that measured the level of environmental exposure to nitrous oxide in the dental field have shown higher values than those detected in the present study [6, 7, 10, 32, 33].

The presence of scavenging systems for anesthetic gases is essential to control and minimize the exposure. Moreover, in order to reduce the risk of nitrous oxide exposure in outpatient settings, a ventilation system is necessary to guarantee a sufficient number of air changes/hour, as well as adequate gas evacuation systems. On the basis of the results of the bibliographic research on the recommended values, further tests should be performed to ensure the correct number of at least 6–7 air changes/hour in the area where the anesthetic agents are used, in accordance with the minimum limits proposed by one of the cited legislation [27].

Although the concentrations in urinary samples are normal if compared with the reference values, the results of the study show a strong correlation between the environmental nitrous oxide and urine concentrations of HCWs, mainly dental assistants, involved in the dental ambulatory sessions. Such result highlights even more the important role of the scavenger system in order to maintain the environmental concentration at a low level and, therefore, minimize the level of exposure of HCWs. Socio-demographic variables of the patients (age and gender) show weak negative correlations with environmental nitrous oxide concentrations. Reducing the age and the percentage of male patients involved in the dental sessions corresponds to a weak increase of environmental nitrous oxide concentrations without any statistical significance.

However, this pilot study has some limitations in the overall assessment of the risk of exposure to nitrous oxide for the personnel working in dental ambulatories, mainly due to the small sample size of the monitored dental interventions. Other studies are necessary in order to confirm the results of the present retrospective pilot study and, above all, complete the evaluation of the calculation algorithm on the number of effective air changes necessary to reduce the risk of exposure in dental ambulatory settings.

As regards the use of nitrous oxide in dental procedures, other studies were carried out on dental extractions, dental restorations and sealant, surgical placement of dental implants with the use of a single or double mask system [2, 7, 8, 34].

The use of nitrous oxide-sedation is a safe and effective option also for patients undergoing digestive endoscopy procedure [35–37] and other painful and minor surgical procedures in children, such as venipuncture, intravenous cannula placement, lumbar puncture, bone marrow aspiration, laceration repair, dental care, and minor dermatologic procedures in pediatric field [38–41]. In both dental and other medical procedures, the environmental concentrations are lower in the presence of a double mask system compared to the single mask system. Some studies have shown that the introduction of scavenging systems can reduce anesthetic gas pollution by over 90% [5, 42–44], as demonstrated also by the present study. More rarely, urinary concentrations in the exposed personnel were detected by other studies [39].

Other previous surveys reported some similar methods of measurement of environmental nitrous oxide concentrations [2, 4, 5, 7], while others indicated different ones [4, 8–10, 41].

## Conclusions

The results of the present study contribute to the need to deepen and implement technical standards, criteria and systems, implant and structure requirements, as well as clinical-anesthesiological analyses useful to assess and define the advantages and disadvantages of the different analgesic/sedation systems. This study has the aim of protecting both the patients’ safety and personnel exposure conditions that, as of today, are not yet completely defined or assimilated to others contemporaneously binding.

In conclusion, the authors would recommend the relevant role and use of nitrous oxide in a general dental ambulatory, and the good performance of the exhaust system used in the study. The authors intend to highlight the necessity of adopting a gas scavenging system, in an environment with adequate air changes/hour, and specific methodological indications in the anesthesia procedure to reduce the risk: correct mask size and adequate pressure of adhesion to the patient’s face, reduction of the time of administration and dilution of gas concentration, etc. Therefore, in the presence of several recommendations without specific regulatory limits for diagnostic and/or therapeutic procedures that use nitrous oxide in non-operating room environments, the authors intended to give a contribution to the ongoing discussion on the definition of the reference values of environmental concentration of nitrous oxide and air change/hour necessary for the specific setting.

## Abbreviations

ACGIH: American Conference of Governmental Industrial Hygienists
AIA: Academy of Architecture for Health
ANSI: American National Standards Institute
ASHRAE: American Society of Heating, Refrigerating and Air-Conditioning Engineers
DCA: Decreto del Commissario ad Acta (Commissioner ad Acta Decree) – Regional Authority
HCWs: Health Care Workers
ICSC: International Chemical Safety Cards
ISPESL: Istituto Superiore per la Prevenzione e la Sicurezza del Lavoro (High Institute for the Prevention and Safety of Work
NIOSH: National Institute for Occupational Safety and Health
REL: Recommended Exposure Limit
SD: Standard Deviation
TLV: Threshold Limit Value
TWA: Time-Weighted Average
